# LONGITUDINAL MENTAL HEALTH CONSEQUENCES OF PHYSICAL DISABILITIES: THE MEDIATING ROLE OF PERCEIVED DISCRIMINATION

**DOI:** 10.1093/geroni/igz038.922

**Published:** 2019-11-08

**Authors:** Eun Ha Namkung, Deborah Carr

**Affiliations:** 1 Brandeis University, Waltham, Massachusetts, United States; 2 Boston University, Boston, Massachusetts, United States

## Abstract

Individuals with disabilities have been historically mistreated by discrimination. The detrimental mental health effects of self-reported interpersonal discrimination are well established. However, little empirical attention has been given to the role of perceived discrimination in the adverse mental health outcomes of adults with physical disabilities. This study aims to examine whether daily interpersonal discrimination (i.e., microaggression) mediates the prospective association between having a functional impairment and subsequent changes in the individuals’ mental health outcomes over their midlife and old age. To address this question, this study used data from two waves of a population-based national study, the National Survey of Midlife Development in the United States, covering a 7- to 9-year period (n= 2,503; Mage at baseline = 57, SDage = 11). Physical disability or functional impairment was assessed with items adapted from the SF-36, capturing difficulty with nine activities of daily living. Having functional impairment at the baseline assessment was associated with increases in depressive symptoms and negative affect over the study period. Daily interpersonal discrimination partially mediated this longitudinal association, explaining 7.4% (for depressive symptoms) to 8.1% (for negative affect) of the total effects. Exposure to discrimination and its mental health consequences were also more pronounced at younger ages. Disability-related perceived discrimination is an under-recognized mechanism that is likely to contribute to mental health inequities in later life. Professionals in health and disability policy, research, and practice need to concentrate efforts on developing policy and programs that reduce discrimination experienced by US adults with disabilities.

